# Factors affecting fledglings survival in urban population of European blackbirds in Szczecin (NW Poland)

**DOI:** 10.1038/s41598-023-46027-w

**Published:** 2023-10-31

**Authors:** Dariusz Wysocki, Marta Witkowska, Szymon Walczakiewicz

**Affiliations:** 1 Institute of Marine and Environmental Sciences, University of Szczecin, Szczecin, Poland; 2 Ornithology Unit, Department of Vertebrate Ecology and Zoology, Faculty of Biology, University of Gdańsk, Gdańsk, Poland

**Keywords:** Ecology, Zoology

## Abstract

The first-year survival alters population growth rates and viability in birds, however this period remains the least-studied of the avian life stages. Here we present results of the 19 years of study of fledglings apparent survival of urban population of European blackbird *Turdus merula* in Szczecin (NW Poland). We checked for possible influence on survival of several factors, including parental traits, such as parental age, their previous breeding experience, natal brood size, presence of another brood in a given breeding season and the time gap between clutches of a particular pair. Moreover, we incorporate into our analysis fledging’s hierarchy in the brood, its fledging time in the breeding season, temperature and precipitation during the first months of life. We also investigated changes in the apparent survival over 19 years. We found that the individual’s hierarchy in the nest, and the day of fledging had the strongest influence on the apparent survival, with heavier birds fledged earlier in the season surviving better. Increase in parental age and previous breeding experience of the pair could result in enhanced survival. Surprisingly increased precipitation lowered fledglings’ survival. During the 19 years of the study fledglings’ apparent survival dropped about 10%.

## Introduction

Population growth rates and viability in birds are usually sensitive to first-year survival^1–3^, but because of the difficulty in tracking fledglings of most bird species, this period remains the least-studied of the avian life stages. Juvenile survival is also often found to be more sensitive than adult survival to fluctuations in environmental conditions, including weather, habitat structure and population density, so the post-fledging period is believed to represent a time of intense selective pressure^4,5^. The majority of post-fledging mortality in passerines occurs during the first 3 weeks after fledging^3^, when they are parent-dependent and have to develop foraging and predator-avoidance skills^6^. Because poor locomotor efficiency can affect the ability to escape from predators, some species of songbirds like the Lark Bunting *Calamospiza melanocorys*^7^ lose as many as 70% of their young, with mortality being particularly high in the first few days after fledging^8^. External factors like parental quality, habitat quality and environment can also be important predictors of annual apparent survival and recruitment, and each of these can be manifested differently across the years^9,10^ Parents can reduce the impact of environmental variability on progeny survival by extending the brooding period when temperatures are low or increasing their food-seeking efforts, but their buffering abilities are restricted^11^. Evidence of delayed parental effects on offspring survival until independence in wild populations remains limited to date, as high-quality longitudinal datasets with known individuals are rare^11,12^. During the last 50 years, global temperatures have risen by about 1 °C^13^. Higher temperatures and changes in precipitation patterns have resulted in changes in the conditions that animals experience on their breeding grounds^14,15^. In the case of songbirds, temperature and precipitation are closely related to the availability of invertebrate prey^16^ , but on the other hand, earlier breeding increases the possibility of failure following sudden weather changes in early spring^17^ as well as possible mismatches between food availability and the need to raise offspring successfully^18^ . Hence, it is extremely important to know how these changes and parental skills impact the fledglings’ survival.

Here we present the results of a 19-year (2002–2020) study of an urban population of the European Blackbird *Turdus merula* conducted in Szczecin (NW Poland). The European Blackbird (henceforth: Blackbird) is a model species with a well-known biology^19,20^, but fledgling survival has only been studied once, by Magrath^21^, who used fewer potential survival predictors compared to our study. Compared to other urban populations studied in Europe, productivity in the target population is low^22^ , with breeding success, as well as some other life-history traits, impacted by the Blackbirds’ age^23–28^. Our earlier studies^29,30^ indicated that a Blackbird’s reproductive performance peaks at 5–6 years old and then declines as senescence sets in, which potentially could impact the fledglings’ survival. In this study, we were able to test whether the same pattern was observed in the case of fledgling survival until independence. The main aim of this work was to gain insight into the influence of multiple natal factors (affecting the early phase of life) on the survival of fledglings until independence.

## Materials and methods

### Data collection

The data for this study were gathered during research on the ecology of an urban population of Blackbirds from 2002 up to and including 2020 in the Stefan Żeromski Park, Szczecin, NW Poland (53°260′ N, 14°330′ E). Situated in the city centre, this park (area 21.9 ha) is surrounded by streets and buildings. During the study period, no parts of the park or its neighbourhood were resurfaced with concrete or asphalt. Occasionally, fallen trees and some shrubs were replaced with new ones. The Blackbird is considered to be a socially monogamous species with biparental care. In the studied population, the social mating system ranges from monogamy to polygyny^31^. Breeding pairs have up to three successful broods (exceptionally four). Hatching is highly asynchronous^32^, nestlings stay in the nest for 14 days^20^, and for another 2-3 weeks as fledglings they are dependent on parental feeding^33^. The density of the population ranged from 1.9 to 2.2 pairs/ha (average 1.9 ± 0.4). The average number of pairs was 51 ± 11.9 (range 34–70), the average number of broods per pair was 2.1 ± 0.4 (range 1.4–2.7), and the breeding success of 2221 broods was 26.1 ± 8.5% (range 3–39%). During the breeding season, an average pair had 1.7 ± 0.6 fledglings (range 0.2–3.1). Over 90% of the breeding population (captured by mist net, or ringed in the nest as a nestling) were marked with a metal ring and additionally, with combinations of four colour rings enabling individual identification without the need for re-capture^34^. Nestlings were ringed between the 5th and 9th day of life. The trapping, ringing and marking of the birds were supervised by Dariusz Wysocki (ringing licence No. 390/2018 from the Polish Academy of Sciences). During the breeding season (March–August), 1–3 persons using binoculars surveyed the study area (21.9 ha) for 4–8 h every day to locate the pairs’ territories, track their nests, and to confirm the survival of the fledglings by resighting colour-ring combinations on individuals present in the study site.

As the independent fledglings hardly ever dispersed from the park during the first 45 days of their life (the youngest fledgling outside the park was observed at the age of 62 days), we believe that the impact of dispersal on our results is negligible.

Previous studies on this urban population of Blackbirds showed that weather parameters such as precipitation and temperature measured in the 30 days prior to the clutch initiation day influenced the beginning of the breeding season^29^. Therefore, we decided to use the same 30-day period to describe the weather conditions that might influence fledgling survival. The temperature and precipitation data during the first 30 days of life of a particular fledgling were obtained from the weather station in Szczecin (53°23'43'' N 14°37'22'' E) [Source: Institute of Meteorology and Water Management-National Research Institute (IMGW-PIB)]^35^.

### Data analysis

The analysis was done for fledglings at least 14 days old. All nestlings that left the nest before this age, a frequent occurrence following a predator attack on the nest, were excluded from the analysis. We established an encounter history for each bird, with an individual’s absence in a given week coded as 0, and encountering an individual in a particular week coded as 1^36^. All individual encounter histories started with 1, indicating the presence of the bird in the nest, in its last week before fledging, and later accounted for 7 more resighting occasions. The interval between resighting occasions was 1 week, so that our encounter histories represented 7 weeks of the bird’s life. We included only one resighting of an individual per week, ignoring multiple resightings during one week if they occurred. For each encounter history we established eleven independent variables characterizing a given individual; these we grouped into three categories of traits: (1) fledgling traits—hierarchy in the brood, hatching time in the breeding season, the presence of another brood in a given breeding season, the time interval between a particular pair's clutches, natal clutch/brood size, (2) parental traits—parental age, pair-bond duration of the parents (pair-bond, i.e. pairs staying together in the previous breeding season were assumed to be trained, whereas pairs that first bonded in a given year were treated as non-trained),and (3) environmental traits—weather conditions (precipitation and temperature) during the first months of life.

**Figure 1:**
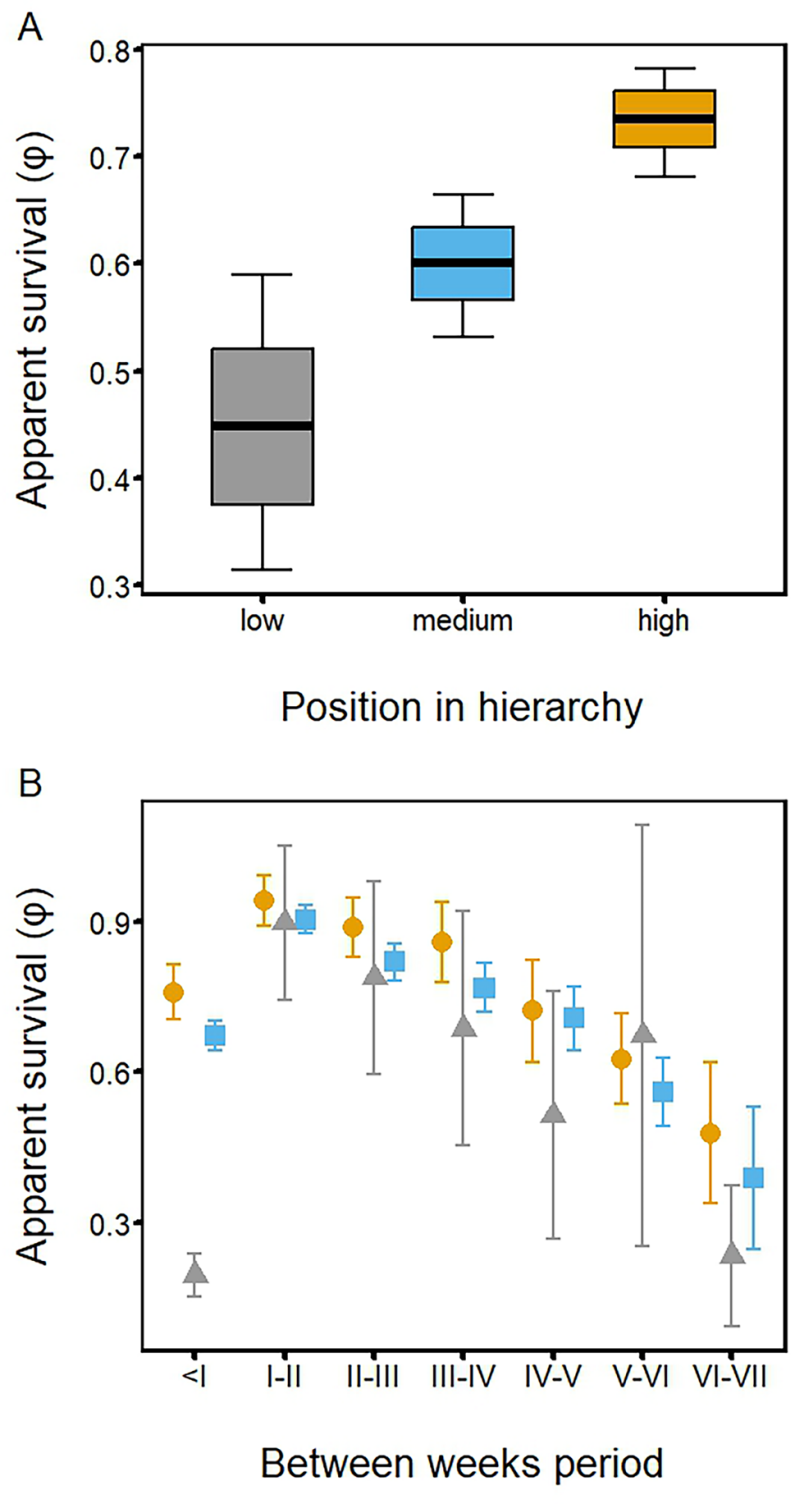
Influence of position in the nest hierarchy on the apparent survival of Blackbird fledglings in NW Poland: (**A**) differences in mean apparent survival between three categories of nest hierarchy. Line-mean value of apparent survival, box-unconditional standard error, whiskers-95% confidence interval; (**B**) changes in apparent survival during 8 weeks of a fledging’s life for three different hierarchy categories (high—yellow dot, medium—blue square, low—grey triangle). Dot—mean value; whiskers—95% confidence interval.

Variables were included together in the same model based on their biological significance in all possible combinations. However, before developing our models, we checked for the collinearity of individual variables with each other and excluded variables with statistically significant Pearson correlation coefficients > 0.25 or < -0.25 together in the same model (Fig. 1S). Instead of disregarding the correlated variables entirely, they were incorporated into separate models, allowing for exploration of their individual effects. All the independent variables are described in detail in Table 1. Although some of the variables, such as ambient temperature or precipitation, could change within the course of the studied 7 weeks, we did not use them as time-varying individual covariates. In total, we were able to obtain 996 individual encounter histories with a full set of independent variables (Table 1S), based on which we estimated apparent survival (φ) and resighting probability (p) using Cormack–Jolly–Seber (CJS) models for live-encounter data. The apparent survival (φ) reflects the chance of a particular individual to survive between established, consecutive instants (e.g. in this study between week_i_ and week_i+1_), but does not distinguish between real mortality and permanent emigration. The resighting probability (p) reflects the probability that a given individual that is present in the study site at a given instant will be detected. All calculations were made using the MARK program with the RMark package in R as its interface^36,37^.

**Table 1:** List of independent variables used in Cormack–Jolly–Seber models for the apparent survival of fledglings in an urban population of the common blackbird breeding in NW Poland.

Group	Variable code	Description	Type
Fledgling’s traits	Hie	Hierarchy of an individual in the nest, established on the basis of weight in the nest	Categorical variable (3 categories: low, medium, high) High—the heaviest chicks in the nest, where the difference in weight between individuals is < 10%; medium—chicks where the weight difference between the lightest chicks in the highest hierarchy category is > 10%, low—chicks where the weight difference between the lightest chicks in the medium category is > 10%
Parental traits	brd.exp	Previous breeding experience of partners in a given pair	Binary variable (0—the first breeding season in the pair; 1—paired in at least one previous breeding season)
	f.age	Age as the calendar year of a female’s (mother’s) life	Numerical variable
	m.age	Age as the calendar year of a male’s (father’s) life	Numerical variable
	nxt.brd	Occurrence of a next brood of the pair in the breeding season	Binary variable (0—no next brood; 1—pair with an additional brood in the breeding season)
	t.gap	Number of days between the broods of a particular pair in one breeding season	Numerical variable
	brd.size	Number of chicks in a given brood	Numerical variable
Environmental traits	Day	Day of the breeding season when the nestling fledged	Numerical variable (1 = 1st of May)
	Year	Year of the breeding season	Numerical variable
	Tmp	Ambient temperature established as the mean temperature during the 30 days prior to the fledging date	Numerical variable
	Prc	Mean precipitation as the mean amount of rain during the 30 days before the fledging date	Numerical variable

We set up our starting model to include the time-dependent apparent survival and the time-dependent probability of a re-encounter (φ (t) p (t)) and later developed more complex candidate models incorporating a different set of independent variables in order to establish their influence on the fledglings’ apparent survival with both additive and interactive effects. We always assumed a time-dependent probability of re-encounter (p), as the preliminary analysis showed a better fit to the data with such an approach. We used the basic, starting model as our global model, to test for possible overdispersion. For that purpose, we ran the parametric bootstrap goodness of fit test available in MARK, with 1000 run simulations to assess the global model’s fit to the data, calculating the variance inflation factor ĉ as observed ĉ divided by the mean ĉ from simulations. With this approach, we achieved ĉ = 1.26, indicating a slight overdispersion of the global model. However, as values of ĉ < 3 are indicators of overdispersion at an acceptable level, we assumed our global model to be a good starting point^36^. Nonetheless, we corrected for the obtained ĉ using the quasi-Akaike Information Criterion (QAIC_c_) and Akaike weights (w_i_) of each model to rank our set of models, considering the model with the lowest QAIC_c_ and highest w_i_ to be the most informative^38^. We accounted for possible model selection uncertainty by employing full-model averaging of all models with w_i_ > 0.001 to estimate apparent survival and re-encounter probabilities together with unconditional standard errors and 95% confidence intervals. Lastly, for the same reason of model selection uncertainty, we derived the relative variable importance (RVI) based on Akaike weights of candidate models with w_i_ > 0.001 in order to assess the magnitude of the effect of individual covariates on averaged model estimates.

## Results

The three top-ranked candidate models achieved ΔQAIC_c_ < 2, indicating their similar parsimony. Moreover, the Akaike weights did not clearly support just one model: this revealed considerable model selection uncertainty, justifying the approach that derives modelled estimates from full-model averaging (Table 2). Of all the proposed individual covariates included within the model, the ones having the strongest influence on apparent survival, with the same relative variable importance score (RVI = 0.99), were the fledgling’s position in the nest hierarchy, and this covariate’s interaction with time, as well as the consecutive day of the breeding season when fledging took place. Parental age, with the female’s age being relatively more important than the male’s age (RVI = 0.43 and RVI = 0.23 respectively), was of moderate importance, and the breeding experience of the pair yielded low values of this parameter (RVI = 0.09). Two environmental variables were similar in terms of variable importance: precipitation (RVI = 0.24) and year (RVI = 0.29). We found that the effect of brood size, the occurrence of a next brood, the time gap between broods, and temperature, which were not included in the set of models with w_i_ > 0.001, suggesting their negligible impact on an individual’s apparent survival.

**Table 2:** Ranking of candidate models for the apparent survival of fledglings in an urban population of the common blackbird breeding in NW Poland, with w_i_ > 0.001.

No	Apparent survival models	QAIC_c_	ΔQAIC_c_	w_i_	k	Dev
1	φ (t * hie + day + m.age)	996.73	0	0.241	30	932.81
2	φ (t * hie + day)	997.83	1.10	0.139	29	936.17
3	φ (t * hie + day + f.age)	998.40	1.67	0.105	30	934.48
4	φ (t * hie + day + m.age + year)	998.79	2.06	0.086	31	932.60
5	φ (t * hie + day + prc + m.age)	998.95	2.22	0.079	31	932.75
6	φ (t * hie + day + year)	999.16	2.43	0.071	30	935.24
7	φ (t * hie + day + brd.exp)	1000.07	3.34	0.046	30	936.14
8	φ (t * hie + day + prc)	1000.09	3.36	0.045	30	936.16
9	φ (t * hie + day + f.age + year)	1000.32	3.59	0.040	31	934.12
10	φ (t * hie + day + prc + f.age)	1000.52	3.79	0.036	31	934.33
11	φ (t * hie + day + prc + m.age + year)	1001.04	4.31	0.028	32	932.56
12	φ (t * hie + day + brd.exp + yr)	1001.41	4.67	0.023	31	935.22
13	φ (t * hie + day + prc + year)	1001.43	4.70	0.023	31	935.24
14	φ (t * hie + day + prc + brd.exp)	1002.31	5.58	0.015	31	936.11
15	φ (t * hie + day + prc + m.age + year)	1002.50	5.77	0.013	32	934.02
16	{φ (t * hie + day + prc + brd.exp + year)	1003.68	6.95	0.007	32	935.21

All the proposed models assumed a time-dependent re-encounter probability p (t).

The models with ΔQAIC_c_ < 2 are bolded.

*QAIC*_*c*_ quasi-Akaike’s Information Criterion, *ΔQAIC*_*c*_ the difference in QAIC_c_ between a given model and the top-ranking model, *w*_*i*_ Akaike weights, *k* number of parameters, *Dev* deviance, *φ* apparent survival, *hie* a fledgling’s position in the nest hierarchy, *day* day of fledging, *prc* precipitation, *f.age* father’s age, *m.age* mother’s age, *year* breeding season, *brd.exp* breeding experience of a pair. +, additive relationship, *, interaction between variables.

**Figure 2:**
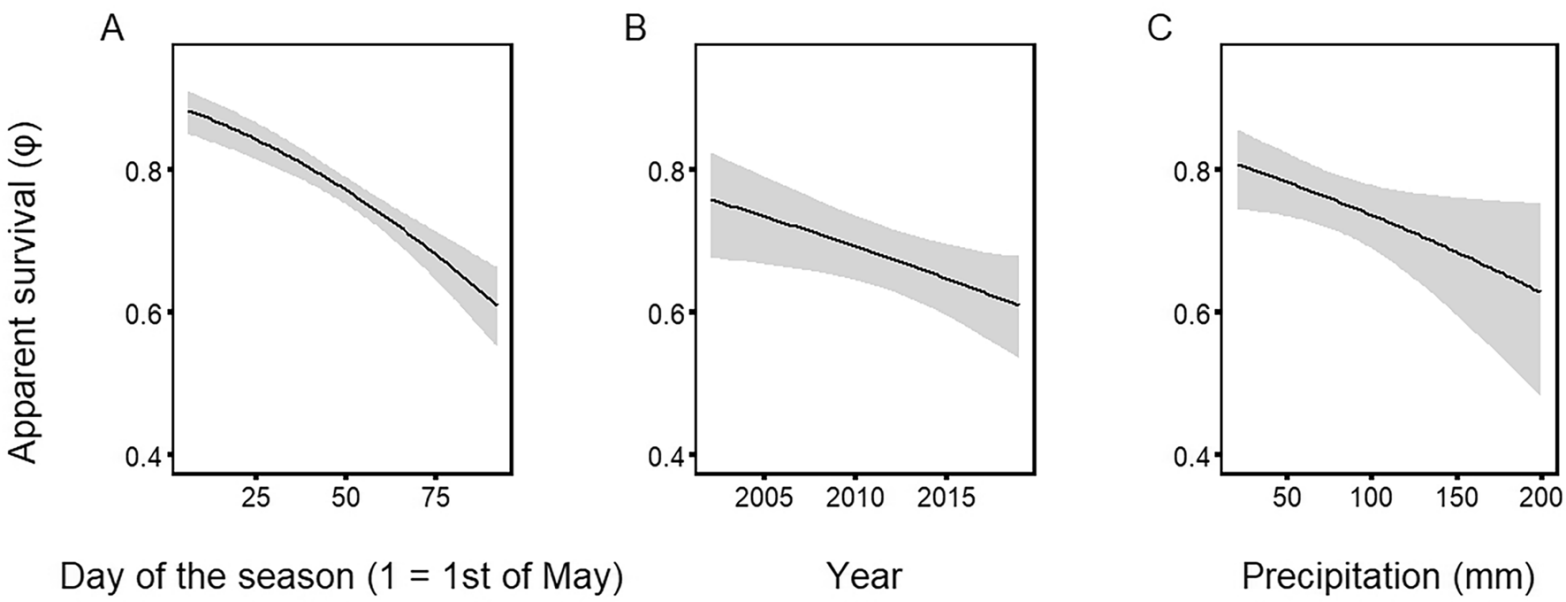
Influence of environmental factors on the apparent survival of Blackbird fledglings in NW Poland: (**A**) day of fledging, (**B**) year, (**C**) precipitation. Black line—modelled relationship between the set of covariates, grey area—95% confidence interval.

**Figure 3:**
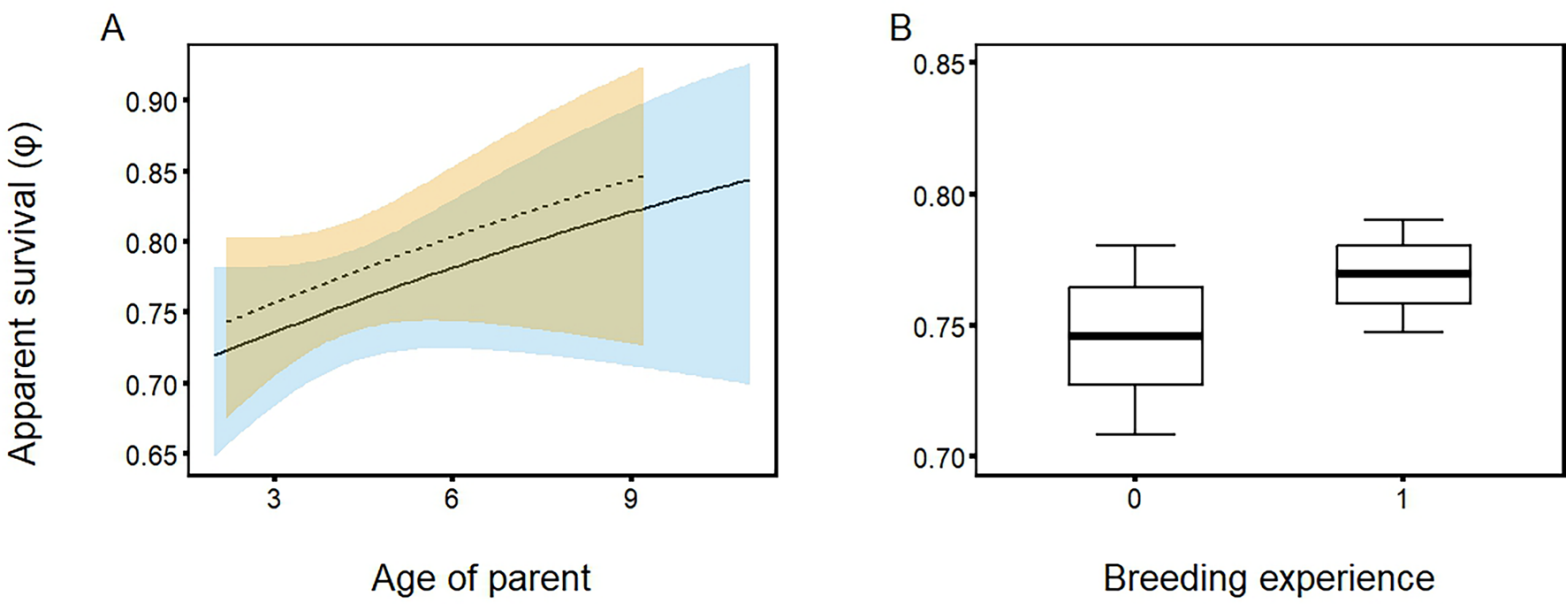
Influence of parental traits on the apparent survival of Blackbird fledglings in NW Poland: (**A**) age of female (mother; dashed line) and male (father; solid line) with a 95% confidence interval [yellow—female (mother), blue—male (father)], (**B**) breeding experience of a pair, where 0—no experience and 1—individuals paired in a previous breeding season. Line—mean value of apparent survival, box—unconditional standard error, whiskers—95% confidence interval.

Both hierarchy and its interaction with time had a large impact on apparent survival, with fledglings higher in the nest hierarchy having greater chances of survival (Fig. 1A). Averaging the model showed that apparent survival changed over the weeks of the study, the lowest values of apparent survival being recorded in the period between leaving the nest and the completion of the first week of life as a fledgling. After the first week, the chances of survival increased rapidly but subsequently decreased moderately in time. When accounting for the effect of hierarchy, the most pronounced difference in apparent survival changes with time was evident in the first period of the fledglings' life outside the nest, when the apparent of survival low-hierarchy birds was lower (mean φ = 0.19) than in birds with medium and high positions in the hierarchy (mean φ = 0.67 and mean φ = 0.75 respectively) (Fig. 1B). After that period, changes in apparent survival with time took a similar course in all three hierarchy groups, with the 95% confidence interval of apparent survival in a given week increasing markedly. The apparent survival of fledglings decreased in time expressed as the day of fledging (Fig. 2A) and the year of the breeding season (Fig. 2B). This parameter decreased with increasing precipitation (Fig. 2C). The chances of a fledgling’s survival increased with the age of both parents (Fig. 3A), as well as the pair’s breeding experience in previous breeding seasons (Fig. 3B).

## Discussion

Our study confirms that hierarchy in the brood and time of fledging are the most important factors governing the survival of fledglings until independence. Natal body mass is a key predictor of viability and fitness in many animals (see^39^ for review). Moreover, in the first study of the factors affecting Blackbird nestling and fledgling survival, Magrath^21^ stated that nestling weight was the most important factor. Because in our study the brood hierarchy was determined on the basis of nestling weight, our results confirm those of Magrath. Our results suggest that the lightest, low-hierarchy fledglings have the least probability of survival compared to medium- and high-hierarchy individuals with substantially higher body weights, and this difference is most pronounced in the first week after leaving the nest. If the fledglings survive the subsequent weeks, the hierarchy differentiates individual survival to a lesser extent, but even after reaching independence in the fifth week of life, the survival of the lightest fledglings seems to be lower than that of the rest of the brood. The observed decrease in apparent survival, with confidence intervals increasing for estimates in consecutive weeks after independence has been reached in the 2–3 weeks outside the nest, could be an effect of better mobility and the lower detection rate of young birds. Moreover, although dispersal from the breeding area in the studied period of fledgling life does not appear to occur frequently, we cannot fully exclude the possibility that dispersal is responsible for the observed gradual decrease in survival probability over time, as CJS apparent-survival models do not distinguish mortality from permanent emigration. Even though the youngest fledgling was observed beyond the park at the age of 62 days, we cannot rule out the possibility that in some cases dispersal could have happened earlier. We are convinced that because of the unfavourable environmental conditions in the vicinity of the park (little greenery, a high density of cats), young Blackbirds very rarely leave the park. The unpropitious conditions for Blackbirds in the park’s surroundings are confirmed by numerous observations of Blackbirds nesting nearby but feeding their young in the park.

The time of hatching strongly impacts the condition and survival of many bird species^40^, including this population of Blackbirds^22^. Offspring that fledge earlier in the season may benefit from milder environmental conditions, more plentiful food, reduced intraspecific competition for resources, as well as lower predation rates and parasitism (see^41^ for a review). It is also possible that the higher apparent survival of fledglings at the beginning of the breeding season is an effect of differences in parental investment between early and late breeders: early chicks may receive a greater investment from their high-quality parents^42^. The day of fledging correlates both with temperature and the occurrence of the next brood, which is why we did not include these three factors together in the same model so as to avoid multicollinearity. Although temperature and the occurrence of the next brood were not indicated as important variables in terms of affecting the apparent survival of fledglings, the effects of all of these factors may act in combination with the day of fledgling. In other words, the day of fledgling masks the effects of temperature and the occurrence of the next brood.

The most unexpected result obtained in this study was that precipitation during the first 30 days of life is negatively related to fledgling survival. Food availability is the most important factor impacting fledgling survival^6,11^, but because precipitation affects food availability for Blackbirds^21,22,43,44^, this result did not support our hypothesis. Similar results were obtained for northern wheatears (*Oenanthe oenanthe*), where rainfall during parental care reduces not only fledgling survival but also their recruitment probability. However, northern wheatears are more dependent on insects, so rainfall significantly reduces food availability for nestlings and fledglings^45^, whereas Blackbirds mostly feed their young with earthworms, the availability of which as a food resource should increase with soil moisture mediated by precipitation. Since higher precipitation increases food availability, one may expect that the time gap between successive broods in the breeding season should be smaller. This shortens the time of care provided by both parents, and could in effect negatively impact the fledglings’ survival. However, we have found that the occurrence of the next brood in a particular breeding season as well as the time gap between successive broods has no effect on fledgling survival, so such a possibility can be ruled out. Another explanation is that rainfall might reduce the time that adults can invest in feeding their fledglings, increasing the possibility of starvation. Moreover, rainfall increases the energy demands of the fledglings^46^, and parents cannot always satisfactorily fulfil them^45,47^. The opposite situation is known in the Mediterranean environment, where low thermoregulatory abilities were probably responsible for the poorer survival of great tit *Parus major* fledglings at higher temperatures^48^. Moreover, hungry fledglings beg for food loudly, and such conspicuous behaviour could increase the risk of predation^49^, especially during the first days after fledging (Wysocki, own observ.).

Blackbirds increase their foraging efficiency at least during the first year of life^23^; parental skills, too, at least in females, continue to improve until the fifth year of life^30^, so one may expect that parental skills acquired with age should have an impact on their progeny’s survival. In this study, we found a moderately positive effect of parental age on the fledglings' apparent survival, but its relative significance compared to the hierarchy in the brood and time of fledging was low. We know that in the studied population, the pair bond duration impacts the start of the breeding season^29^, so it, too, may be expected to affect fledgling survival, but as with parental age, its positive impact on the fledglings’ apparent survival was relatively low.

The presence of another brood in the breeding season has no impact on fledgling survival. In the first part of the breeding season, most pairs abandon their first brood fledglings as soon as the second brood hatchlings appear in the nest. Not feeding their previous brood any longer should imply a higher mortality among those young birds. The results of an earlier study on fledgling adoption^33^ strongly suggest that an intergenerational conflict is taking place, but even with a large data set to hand, we failed to find any next-brood effect on fledgling survival. Furthermore, the good condition of parents may to some extent buffer any possible adverse impact of the next brood. The good condition of females is usually correlated with male condition^50^, and males in good condition can manage to feed the whole brood alone until the next-brood nestlings appear, which is usually due to the acquired independence of the previous fledglings (own observ.).^.^

Caring for more nestlings should be less effective than caring for fewer, but we did not find any effect of brood size on the fledglings’ apparent survival. This is most probably explained by the masking effect of brood hierarchy. Bigger broods usually have a clearly defined hierarchy of nestlings owing to more the pronounced asynchrony of hatching, which occurred less often in broods smaller than four (own observ.), and a nestling's body weight, as expressed by its position in the brood hierarchy later determines its survival as a fledgling.

The most worrying result of this study is the significant decrease in the apparent survival of Blackbird fledglings during the 19 years of this study. We were unable to identify any factors directly responsible for this effect. In contrast to a study done on House Sparrows *Passer domesticus*^51^, we can exclude the possible impact of park reurbanization, because there was no resurfacing with concrete or asphalt in the park during the study period. The level of pollution seems to be stable. For instance, an earlier study on the impact of lead on Blackbird biology^52^ showed that lifetime breeding success decreased with the lead concentration but that this did not affect Blackbird longevity. Moreover, the availability of less nutritious anthropogenic food to Blackbirds, also attractive to their predators^53^, seems to be stable. Changes in the weather conditions are unlikely to have any effect, as no clear trends in temperature and/or precipitation emerged during this time, at least not during the period when the fledglings were dependent on their parents. Corvid predation is not likely to be responsible either, as the numbers of pairs of Carrion Crow *Corvus corone*, Eurasian Magpie *Pica pica*, Eurasian Jay *Garrulus glandarius* and Eurasian Jackdaw *Corvus monedula* do not follow the observed fledgling survival pattern after the increase from 1997 (7 pairs) to 2006 (19 pairs), the number of pairs was stable in 2007–2010 (16–19 pair), but later decreased (15–11 pairs). In addition, we have no data on the numbers of Beech Martens *Martes foina*, Domestic Cats *Felis catus*, Domestic Dogs *Canis familiaris* or Foxes *Vulpes vulpes* which regularly hunt in the park, but because these animals were regularly sighted ever since the start of this study, any impact on their part decreasing fledgling survival over the years must have been similar throughout the study period. Although we did not note any trend in temperature and precipitation changes during that period, the increasing frequency of extreme weather events in recent years, such as long periods of drought and heavy rains^54^ , may have reduced the water available for plants, especially in cities, with the consequent deterioration in the condition of the vegetation^55^. This could affect food availability and therefore the survival of Blackbird fledglings.

In summary, we found that several of the studied factors influence the apparent survival of Blackbird fledglings, with hierarchy in the brood and time of fledging being relatively the most important ones. Individuals with a higher position in the hierarchy had greater chances of survival, especially in the first week after leaving the nest, just as birds fledging early in the breeding season. We pointed out the importance of parental traits, such as the age of both parents and their previous breeding experience, and the positive effect of these factors on fledging survival, an aspect of Blackbird biology not previously studied^21^. Increased precipitation reduces the survival of Blackbird fledglings, but the reason for this is unclear. We also found a significant decrease in fledgling survival during the 19 years of this study, but the factors directly responsible for this are unknown.

### Supplementary Information

Supplementary Information.

## Data Availability

Supplementary data for this article can be accessed online: https://figshare.com/account/items/23822190/edit.
