# Supplementary material for: Factors affecting fledglings survival in urban population of European blackbirds in Szczecin (NW Poland)
Source: Sci Rep. 2023 Oct 31;13:18723. doi: 10.1038/s41598-023-46027-w (PMC10618181; doi:10.1038/s41598-023-46027-w)
Supplement: Supplementary file 1 — Supplementary Information. [file 41598_2023_46027_MOESM1_ESM.docx]

Factors affecting fledglings survival in urban population of European Blackbirds in Szczecin (NW Poland)

Dariusz Wysocki^1*^, Marta Witkowska^2^, Szymon Walczakiewicz^1^,

^1^Institute of Marine & Environmental Sciences, University of Szczecin, Poland

^2^ Ornithology Unit, Department of Vertebrate Ecology and Zoology, Faculty of Biology, University of Gdańsk, Poland

Suplementarny materials

| Table 1S. Summary of number and percentage of resighted individuals during 7 weeks of observations after fledging. | | | | | | | | | | | | | | | |
| --- | --- | --- | --- | --- | --- | --- | --- | --- | --- | --- | --- | --- | --- | --- | --- |
| Year | All ring individuals | I week | | II week | | III week | | IV week | | V week | | VI week | | VII week | |
|  |  | N | % | N | % | N | % | N | % | N | % | N | % | N | % |
| 2002 | 72 | 23 | 32% | 16 | 22% | 13 | 18% | 7 | 10% | 8 | 11% | 6 | 8% | 4 | 6% |
| 2003 | 71 | 33 | 46% | 29 | 41% | 14 | 20% | 13 | 18% | 11 | 15% | 9 | 13% | 6 | 8% |
| 2004 | 42 | 25 | 40% | 15 | 36% | 14 | 33% | 9 | 21% | 7 | 17% | 6 | 14% | 3 | 7% |
| 2005 | 99 | 46 | 46% | 37 | 38% | 30 | 30% | 29 | 29% | 13 | 13% | 8 | 8% | 4 | 4 |
| 2006 | 78 | 34 | 44% | 27 | 35% | 28 | 36% | 10 | 13% | 12 | 15% | 6 | 8% | 4 | 5% |
| 2007 | 56 | 29 | 52% | 21 | 38% | 17 | 30% | 9 | 16% | 6 | 29% | 3 | 5% | 2 | 4% |
| 2008 | 21 | 12 | 57% | 11 | 52% | 6 | 28% | 5 | 24% | 3 | 14% | 3 | 14% | 2 | 10% |
| 2009 | 44 | 11 | 25% | 6 | 14% | 5 | 11% | 6 | 14% | 4 | 9% | 4 | 9% | 3 | 7% |
| 2014 | 57 | 16 | 28% | 17 | 12% | 9 | 16% | 8 | 14% | 5 | 9% | 0 | 0% | 0 | 0% |
| 2015 | 55 | 31 | 56% | 25 | 45% | 17 | 31% | 5 | 9% | 6 | 11% | 2 | 4% | 2 | 4% |
| 2016 | 132 | 41 | 31% | 41 | 31% | 41 | 31% | 25 | 19% | 18 | 14% | 16 | 12% | 9 | 7% |
| 2017 | 98 | 32 | 33% | 41 | 42% | 26 | 27% | 27 | 28% | 12 | 12% | 6 | 6% | 4 | 4% |
| 2018 | 97 | 30 | 31% | 33 | 34% | 22 | 23% | 15 | 15% | 10 | 10% | 4 | 4% | 4 | 4% |
| 2019 | 74 | 30 | 41% | 29 | 39% | 22 | 30% | 19 | 26% | 13 | 18% | 10 | 14% | 5 | 7% |
| Total | 996 | 385 | 39% | 348 | 35% | 264 | 27% | 187 | 19% | 128 | 13% | 83 | 8% | 52 | 5% |


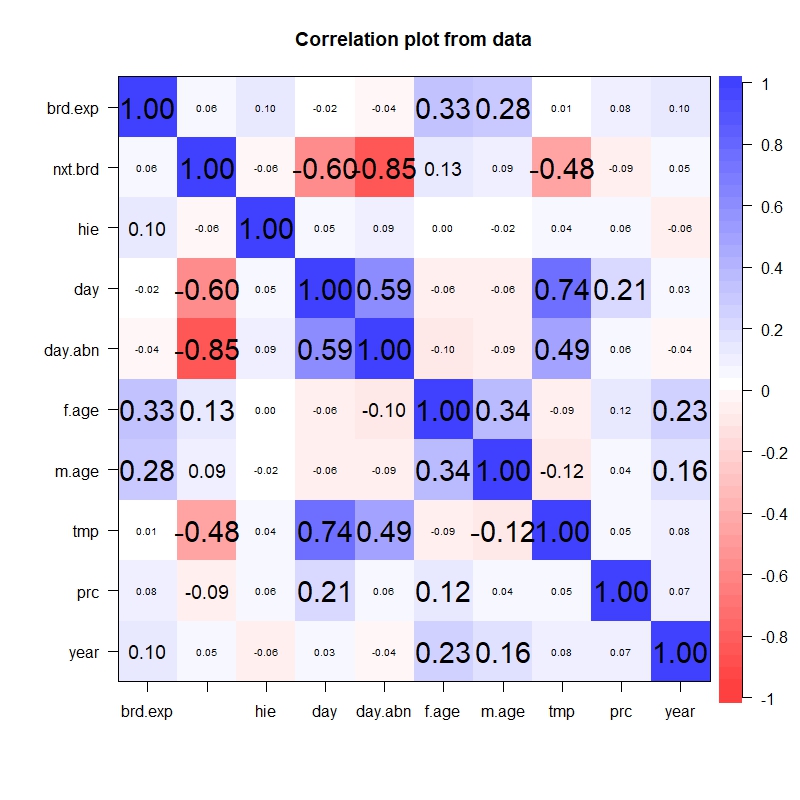
Fig. 1S. Results of Pearson Correlation between variables used in the survival models. Values of the Pearson correlation coefficient ( r ) are given. Large and bolded text indicate statistical significance of the correlation with p < 0.05.
